# Epidemiological Shifts in Children Respiratory Pathogens in Shenzhen, China: A Comparative Analysis Before and After the Relaxation of COVID‐19 Non‐Pharmaceutical Interventions

**DOI:** 10.1111/irv.70114

**Published:** 2025-04-24

**Authors:** Heping Wang, Yuping Guo, Rongjun Wang, Zihao Liu, Li Li, Yuzheng Li, Yanmin Bao, Wenjian Wang

**Affiliations:** ^1^ Shenzhen Children's Hospital Shenzhen Guangdong China; ^2^ Yudu County Traditional Chinese Medicine Hospital Ganzhou Jiangxi China

**Keywords:** acute respiratory infections, children, epidemiology, non‐pharmaceutical interventions, respiratory pathogens

## Abstract

**Background:**

The COVID‐19 pandemic and associated non‐pharmaceutical interventions significantly altered the epidemiology of respiratory pathogens. This study aimed to evaluate the changes in the prevalence and distribution of respiratory pathogens among children with acute respiratory infections (ARIs) before and after the relaxation of COVID‐19 restrictions in Shenzhen, China.

**Methods:**

This study enrolled hospitalized children with ARIs in Shenzhen Children's Hospital during the COVID‐19 epidemic and those post‐epidemic period. Demographic data of the patients enrolled were retrieved from the Shenzhen Children's Hospital electronic patient dossiers. Nasopharyngeal swabs were collected and detected for 11 pathogens, and epidemiological trends were analyzed by age, season, and pathogen distribution.

**Results:**

A total of 40,174 children with ARIs were enrolled, including 14,816 during the COVID‐19 epidemic and 25,358 in the post‐epidemic period. Hospital admissions for ARIs increased by 71.2% in the post‐epidemic period. The median age of patients rose from 27 to 47 months. Pathogen detection rates increased significantly from 59.3% during epidemic period to 73.0% in post‐epidemic period (*p* < 0.001), with co‐detection (≥ 2 pathogens) rising from 10.5% to 21.2%. The dominant pathogens shifted from HRV, RSV, and HPIV during COVID‐19 epidemic to HRV, MP, and RSV in the post‐epidemic. Notably, MP detection rates surged from 1.69% to 20.87%, while RSV and HPIV peaks were replaced by MP and HMPV in winter.

**Conclusion:**

The relaxation of COVID‐19 non‐pharmaceutical interventions led to a significant rebound in ARIs among children, with altered pathogen dominance and increased co‐detection.

**Abbreviations**
InfA: influenza A
InfB: influenza B
HPIV: human parainfluenza virus
HRSV: human respiratory syncytial virus
HAdV: human adenoviruses
HMPV: human metapneumovirus
HRV: human rhinovirus
HBoV: human bocavirus
HCoV: human coronavirus
Ch: chlamydia
MP:
*Mycoplasma pneumoniae*

PCR: polymerase chain reaction

## 1 Background

During the COVID‐19 pandemic, non‐pharmaceutical interventions (NPIs) such as social distancing, travel restrictions, and home‐stay policies significantly impacted the epidemiology of common respiratory pathogens [1, 2, 3, 4]. In late 2022, with the gradual relaxation of these NPIs, the region experienced a surge and peak in COVID‐19 cases from late 2022 to early 2023. This was followed by a significant increase in influenza A cases during the spring of 2023 and a rapid rise in respiratory infection cases during the autumn–winter of 2023 [5, 6, 7]. Globally, several countries also reported unusual increases in cases of common respiratory pathogens, such as influenza viruses (IFV), outside their typical seasons following the relaxation or removal of NPIs after 2022 [8, 9, 10]. Prior to the COVID‐19 pandemic, annual seasonal fluctuations of a range of pathogens, including influenza A (InfA), influenza B (InfB), respiratory syncytial virus (RSV), and *Mycoplasma pneumoniae* (MP), had been systematically documented [11, 12]. Following the implementation of NPIs across various countries to curb the spread of COVID‐19, certain pathogens exhibited episodic outbreaks [13, 14]. In China, the phased relaxation of NPIs began at the end of 2022, which marked a temporal divergence from the approaches adopted by most international jurisdictions.

We have already conducted comparative studies on the epidemiology of common respiratory pathogens before and during the pandemic [15, 16]. We found that the detection rates of InfA, MP, human adenovirus (HAdV), and human rhinovirus (HRV) decreased, while infections with RSV and human parainfluenza virus (HPIV) increased, especially in toddlers following the lockdown.

During the pandemic, the implementation of NPIs led to a reduction in the detection rates of some common respiratory pathogens. However, following the optimization and adjustment of COVID‐19 prevention and control measures, these pathogens may experience outbreaks. The outbreaks of vaccine‐preventable pathogens observed in other countries have significant reference value for the post‐pandemic situation in China [17]. In the past few months, shortly after the relaxation of epidemic prevention and control measures, most respiratory infections were caused by the COVID‐19 Omicron variant, which has influenced the emergence of common respiratory pathogens.

This study aims to compare the detection rates of respiratory pathogens among pediatric inpatients at Shenzhen Children's Hospital during the stable period of epidemic prevention and control and the stable period after the optimization and adjustment of these measures. We investigated whether the detection rates of respiratory pathogens have returned to before pandemic levels or exceeded them after the relaxation of epidemic prevention and control measures. This study provides baseline data for potential future pediatric respiratory infections and offers suggestion for the prevention and treatment of pediatric respiratory diseases.

## 2 Methods

### 2.1 Patients Information

Patients with acute respiratory infections (ARIs) between March 2021 to February 2022 (during the COVID‐19 epidemic) and March 2023 to February 2024 (post‐epidemic period) admitted to the pediatric wards were enrolled in Shenzhen Children's Hospital. The inclusion criteria were as follows: age below 14 years and one or more respiratory symptoms (cough, sore throat, body temperature above 37.5°C, and dyspnea/tachypnoea). The exclusion criteria were as follows: Repeated the detection of the same patient within 1 week, the patient was over 14 years old, and the age or gender information was incomplete. Laboratory and demographic data of the patients enrolled in this study were retrieved from the Shenzhen Children's Hospital electronic patient dossiers. The study protocol was approved and waived informed consent by the Ethical Committee of Shenzhen Children's Hospital (202318002). The study was performed in accordance with relevant guidelines and regulations.

### 2.2 Specimens and Pathogens Detection

Nasopharyngeal swabs were obtained by trained personnel following standard operating procedures within 24 h after admission. The specimens were transported immediately to the laboratory in sterile viral transport media. The total nucleic acids of each specimen were extracted using the EasyPure Viral DNA/RNA Kit (TransGen Biotech, Beijing, China) in accordance with the manufacturer's instructions. Eleven common respiratory pathogens, including InfA, InfB, HPIV, RSV, HAdV, human metapneumovirus (HMPV), HRV, human bocavirus (HBoV), human coronavirus (HCoV), *Chlamydia* (Ch), and MP, were detected using a GeXP‐based multiplex reverse transcription PCR assay [18] (Health Gene Tech., Ningbo, China).

### 2.3 Statistical Analysis

SPSS 21.0 was used to analyze differences in patient number and pathogen prevalence with 95% confidence intervals. Descriptive statistics and paired *t*‐test were applied to describe patient characteristics and difference. Categorical variables were analyzed by Pearson's chi‐squared test. *p* < 0.05 was considered statistically significant.

## 3 Results

### 3.1 Patient Characteristics

**FIGURE 1:**
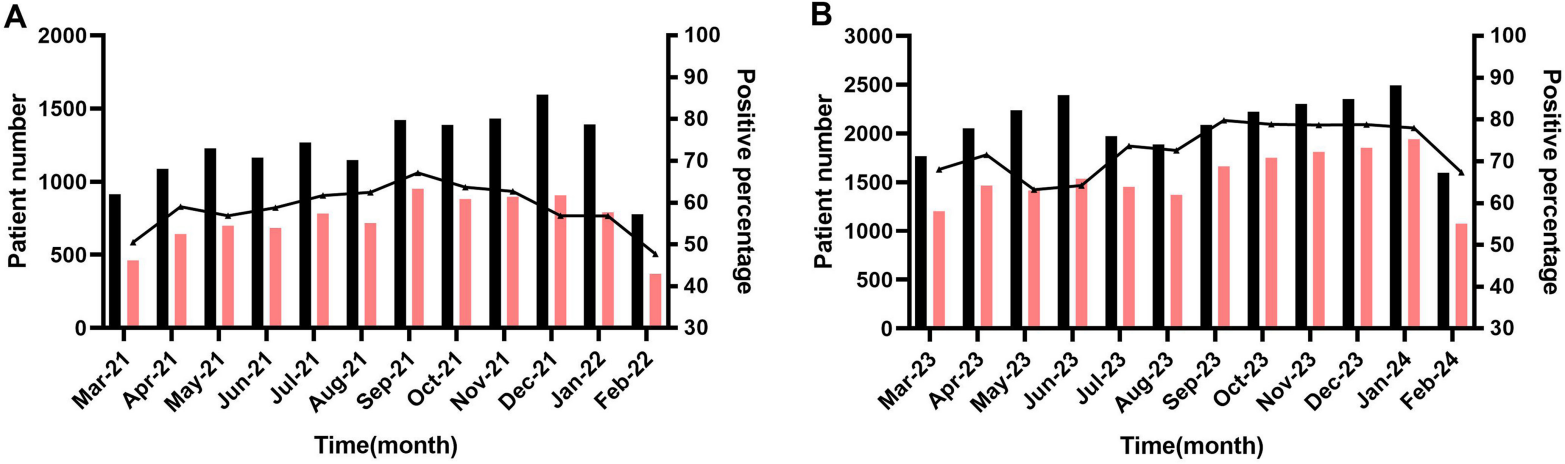
The patient number (black bar) and positive number (pink bar); detection rates of pathogens by month during COVID‐19 epidemic (A) and post‐epidemic (B).

A total of 40,174 children with ARIs were enrolled in this study, including 14,816 during the period from March 2021 to February 2022 (during the COVID‐19 epidemic) and 25,358 during the period from March 2023 to February 2024 (post‐COVID‐19 epidemic). This represents a 71.2% increase in hospital admissions due to ARIs in the post‐epidemic period compared with the epidemic period. The number of monthly hospitalized children for ARIs increased rapidly in the post‐epidemic period (Figure 1). The age of the patients ranged from 1 month to 14 years, with a median age of 27 months during the COVID‐19 epidemic and 47 months in the post‐epidemic period. The patients were divided into four age groups: infant group (1 month–1 year old): 4166 cases during COVID‐19 epidemic and 4972 cases in post‐epidemic; toddler group (1–3 years old): 4353 cases during COVID‐19 epidemic and 5318 cases in post‐epidemic; pre‐school group (3–6 years old): 4078 cases during COVID‐19 epidemic and 6964 cases in post‐epidemic; school‐age children group (6–14 years old): 2219 cases during COVID‐19 epidemic and 8104 cases in post‐epidemic.

The study population included 8765 males (59.2%) and 6051 females (40.8%) during COVID‐19 epidemic (sex ratio: 1.45:1) and 14,761 males (58.2%) and 10,597 females (41.8%) in post‐COVID‐19 epidemic (sex ratio: 1.39:1). There was no significant difference in the sex ratio in both periods (*X* ^2^ = 3.467, *p* = 0.063).

### 3.2 Overall Detection Percentage of the 11 Pathogens

During COVID‐19 epidemic, out of 14,816 specimens, 8779 (59.3%) tested positive for at least one of the 11 pathogens. Among these positive cases, a single pathogen was detected in 7858 (89.5%) patients, while more than two pathogens were detected in 921 (10.5%) patients. In the post‐COVID‐19 epidemic, out of 25,358 specimens, 18,516 (73.0%) detected positive for at least one of the 11 pathogens. Among these, a single pathogen was detected in 14,583 (78.8%) cases, and more than two pathogens were detected in 3933 (21.2%) cases. The detection rate in post‐COVID‐19 epidemic was significantly higher than during COVID‐19 epidemic (*X* ^2^ = 813.523, *p* = 0.001). The rate of detection two or more pathogens was significantly higher in the post‐epidemic period compared with during the epidemic period (*X* ^2^ = 470.719, *p* = 0.001). The most frequently observed co‐detection pattern was MP with HRV, followed by combinations of RSV and HRV, HPIV and HRV, and HAdV and HRV. Notably, MP and HAdV co‐detection also demonstrated significant prevalence.

### 3.3 Changes of Specific Pathogens Between During and Post‐Epidemic Period

The top three pathogens detected during COVID‐19 epidemic were HRV, RSV, and HPIV, while in post‐COVID‐19 epidemic, the dominant pathogens were HRV, MP, and RSV. Five pathogens (InfA, MP, HAdV, HCoV, and HMPV) showed higher detection rates in post‐COVID‐19 epidemic compared with during COVID‐19 epidemic. Notably, the detection rate of InfA increased from 0.06% during COVID‐19 epidemic to 8.01% in post‐epidemic, and MP showed the most significant increase, rising from 1.69% to 20.87%. In contrast, the detection rates of HRV, RSV, HPIV, InfB, and Ch decreased in post‐epidemic compared with during COVID‐19 epidemic. HBoV showed similar detection rates in both periods (Table 1).

**TABLE 1:** Comparison of positive rates of 11 respiratory pathogens during and post‐COVID‐19 epidemic.

Pathogens	During epidemic *N* = 14,816	Post‐epidemic *N* = 25,358	*X* ^2^	*p*
Human rhinovirus (HRV)	4007 (27.05%)	5465 (21.55%)	156.64	0.001
Human respiratory syncytial virus (RSV)	2592 (17.49%)	3085 (12.17%)	218.85	0.001
Human parainfluenza virus (HPIV)	1181 (7.97%)	1677 (6.61%)	26.092	0.001
Human adenovirus (AdV)	424 (2.86%)	2463 (9.71%)	658.13	0.001
Influenza B virus (InfB)	368 (2.48%)	515 (2.03%)	5.245	0.022
Human metapneumovirus (HMPV)	347 (2.34%)	1268 (5.0%)	171.28	0.001
Human bocavirus (HboV)	283 (1.91%)	433 (1.71%)	2.192	0.139
*Mycoplasma pneumoniae* (MP)	251 (1.69%)	5293 (20.87%)	2891.8	0.001
Human coronavirus (HcoV)	185 (1.25%)	531 (2.09%)	38.180	0.001
*Chlamydia* (Ch)	76 (0.51%)	93 (0.37%)	4.773	0.029
Influenza A virus (InfA)	9 (0.06%)	2030 (8.01%)	1225.2	0.001

### 3.4 Changes in Specific Pathogens Based on Season and Age

The detection rates of pathogens in post‐epidemic were significantly higher than during COVID‐19 epidemic across all age groups, with the differences becoming more pronounced as age increased (Table 2). There was no significant difference in seasonal trends during COVID‐19 epidemic and the post‐epidemic periods, with the highest detection rates occurring in autumn in both periods. However, the detection rates were significantly higher in the post‐epidemic period, and the typical decline in detection rates during winter was not observed. Instead, a sustained peak in detection was noted during the winter (Table 2).

**TABLE 2:** Comparison of positive rates of respiratory pathogens based on age and season during and post‐COVID‐19 epidemic.

	During epidemic *N* = 14,816	Post‐epidemic *N* = 25,358	*X* ^2^	*p*
Age groups				
Infant	2610 (62.65%)	3452 (69.43%)	46.64	0.001
Toddler	2942 (67.59%)	4211 (79.18%)	167.2	0.001
Pre‐school	2394 (58.71%)	5326 (76.48%)	386.3	0.001
School	833 (37.54%)	5527 (68.20%)	692.4	0.001
Seasons				
Spring	1803 (55.79%)	4085 (67.45%)	123.628	0.001
Summer	2184 (61.01%)	4326 (69.74%)	77.971	0.001
Autumn	2734 (64.48%)	5225 (79.07%)	271.366	0.001
Winter	2068 (54.96%)	4870 (75.63%)	466.677	0.001

**FIGURE 2:**
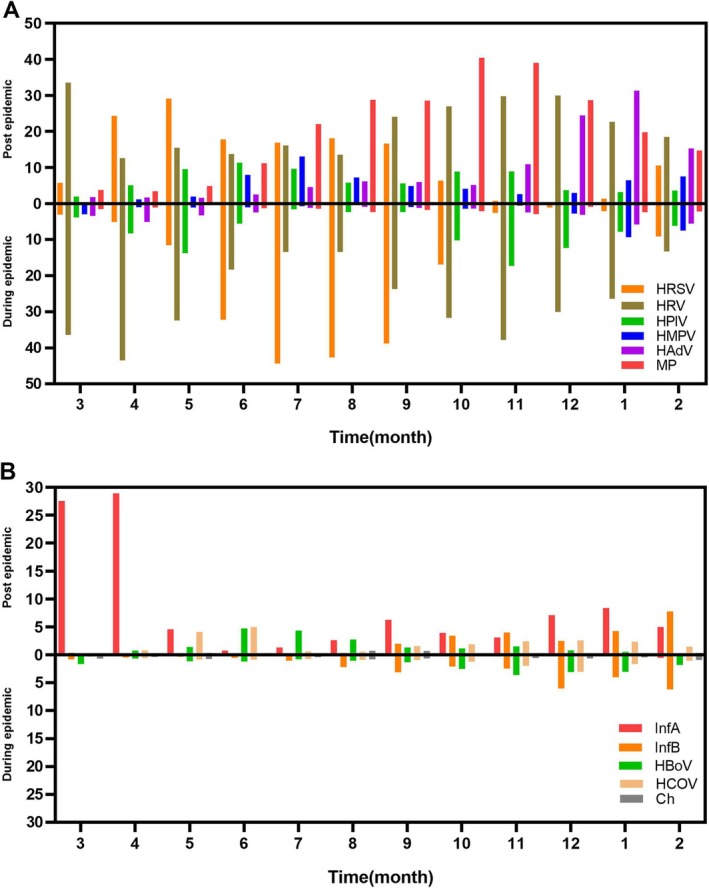
The comparison of detection rate of 11 pathogens by month during COVID‐19 epidemic and post‐epidemic. (A) included six pathogens, HRSV, HRV, HPIV, HMPV, HAdV, and MP; (B) included five pathogens, InfA, InfB, HBoV, HCOV, and Ch.

We also identified some epidemic patterns of viral infections during winter. For example, HAdV showed a peak in detection rates in January, and MP maintained a high detection rate, especially in post‐COVID‐19 epidemic. RSV did not show a significant peak, as its usual peak was replaced by those of MP and HMPV. Similarly, HPIV did not exhibit a significant peak in November as it did during the epidemic period, although its highest detection rate still occurred. This peak was overshadowed by the rise in MP cases (Figure 2).

## 4 Discussion

This study highlights a significant increase in hospital admissions for ARIs in the post‐COVID‐19 period compared with the pandemic period. This surge is likely attributable to the relaxation of public health measures, such as mask‐wearing and social distancing, which were rigorously enforced during the pandemic. The subsequent rise in social interactions likely facilitated the transmission of respiratory pathogens, resulting in a higher incidence of ARIs. Notably, the overall detection rate of respiratory pathogens increased significantly in the post‐epidemic period, with a marked rise in co‐detection of more than two pathogens. Similar trends have been reported globally, with studies from the United States and Europe documenting a rebound in respiratory infections following the relaxation of NPIs [19, 20, 21]. This suggests an increase in co‐infections, potentially due to weakened immunity in children who had limited exposure to common pathogens during the pandemic.

Significant shifts in the prevalence of specific pathogens were observed. HRV remained the most frequently detected pathogen in both periods, but its relative dominance decreased post‐epidemic. In contrast, MP and InfA showed substantial increases in detection rates. These changes may reflect altered pathogen circulation patterns due to shifts in immunocompetence and healthcare‐seeking behaviors, such as increased influenza vaccination coverage and improved hand hygiene habits, which may influence the transmission of respiratory pathogens among children. In post‐pandemic, the influenza A vaccination rate among children in Shenzhen has increased significantly. However, the detection rate of influenza A virus infections did not decline as expected, warranting further investigation into the underlying reasons. The decline in detection rates of RSV and HPIV, along with the absence of their typical seasonal peaks, suggests that the pandemic disrupted the usual seasonal dynamics of these viruses. Instead, MP and HMPV emerged as dominant pathogens in the post‐pandemic period, particularly during winter. Similar increases in MP cases have been reported in Beijing, Hebei, Chongqing, and Hunan in China, and Denmark in Europe, though HMPV prevalence remained relatively stable in Beijing [22, 23, 24, 25]. Comparable findings have been reported in other regions, such as Australia and Japan, where RSV and HPIV peaks were delayed or replaced by other pathogens following the relaxation of NPIs [11, 19, 26].

The study also found that pathogen detection rates were significantly higher across all age groups in the post‐epidemic period. This may be due to the cumulative effect of reduced pathogen exposure during the pandemic, leading to a larger susceptible population among older children. Seasonal trends in pathogen detection shifted, with the highest rates occurring in autumn in both periods. However, the post‐pandemic period saw a sustained high detection rate during winter, deviating from the typical decline observed in previous years. Similar observations have been made in studies from Canada and German, where atypical seasonal patterns and prolonged viral activity were noted post‐NPI relaxation [27, 28]. This pattern suggests prolonged circulation of respiratory viruses, likely driven by the relaxation of public health measures and increased population susceptibility.

This study has several limitations. First, the data were collected from a single institution, which may limit the generalizability of the findings. Second, the post‐pandemic study period is relatively short, and longer‐term surveillance is needed to fully understand the evolving epidemiology of ARIs. Third, the study focused solely on hospitalized children, potentially excluding milder cases managed in outpatient settings. Finally, the study did not account for the impact of vaccination rates or other confounding factors that may have influenced the observed trends. In post‐pandemic, increased in hospital visits may inflate detection rates. However, relative pathogen proportions (for instance, MP vs. HRV dominance) were less likely affected, supporting conclusions about shifting epidemiology.

The findings underscore the need for enhanced surveillance and preparedness for respiratory infections in the post‐pandemic era. The emergence of MP and HMPV as dominant pathogens highlights the importance of incorporating these pathogens into routine diagnostic panels and treatment protocols. Additionally, the observed changes in seasonal patterns suggest that traditional approaches to predicting and managing respiratory infections may require revision. The global experience, as evidenced by studies from multiple countries, further supports the need for adaptive public health strategies to manage the evolving epidemiology of respiratory pathogens [10, 13, 29]. Public health strategies should prioritize vaccination campaigns, particularly for influenza and other preventable respiratory infections, and reinforce hygiene practices to mitigate the impact of ARIs. These measures will be critical in addressing the increased burden of respiratory infections and protecting vulnerable populations, particularly children, in the post‐COVID‐19 epidemic.

## 5 Conclusion

In conclusion, this study highlights significant changes in the epidemiology of ARIs among children following the COVID‐19 pandemic. The increased burden of ARIs, shifts in pathogen distribution, and altered seasonal patterns underscore the need for ongoing surveillance and adaptive public health strategies to address the evolving landscape of respiratory infections.

## Author Contributions

**Heping Wang:** conceptualization, writing – original draft, writing – review and editing. **Yuping Guo:** data curation, formal analysis, conceptualization. **Rongjun Wang:** data curation, visualization. **Zihao Liu:** methodology, software, data curation. **Li Li:** supervision, resources, investigation. *Yuzheng Li1: resources. **Yanmin Bao:** conceptualization, writing – review and editing, project administration, funding acquisition. **Wenjian Wang:** conceptualization, project administration, writing – review and editing.

## Ethics Statement

The study protocol was approved and waived informed consent by the Ethical Committee of Shenzhen Children's Hospital (202318002). All experiments were performed under the relevant guidelines and regulations.

## Consent

The authors have nothing to report.

## Conflicts of Interest

The authors declare no conflicts of interest.

### Peer Review

The peer review history for this article is available at https://www.webofscience.com/api/gateway/wos/peer‐review/10.1111/irv.70114.

## Data Availability

All data generated or analyzed during this study are included in this published article.
